# 2282. Characterizing Antibacterial Regimen Modification, Patient Characteristics, and Outcomes for Patients with Complicated Gram-Negative Bacterial Infections

**DOI:** 10.1093/ofid/ofad500.1904

**Published:** 2023-11-27

**Authors:** Jason Yamaki, Emre Yucel, Alexandre H Watanabe

**Affiliations:** Chapman University School of Pharmacy, Irvine, California; Merck & Co., Inc., North Wales, Pennsylvania; Merck & Co., Inc., North Wales, Pennsylvania

## Abstract

**Background:**

There are few studies describing the rationale or frequency of empiric or directed therapy treatment modification behaviors among patients with complicated Gram-negative bacterial infections (GNBI). We sought to characterize patient and treatment characteristics, details of regimen modification, and patient outcomes.

**Figure:**
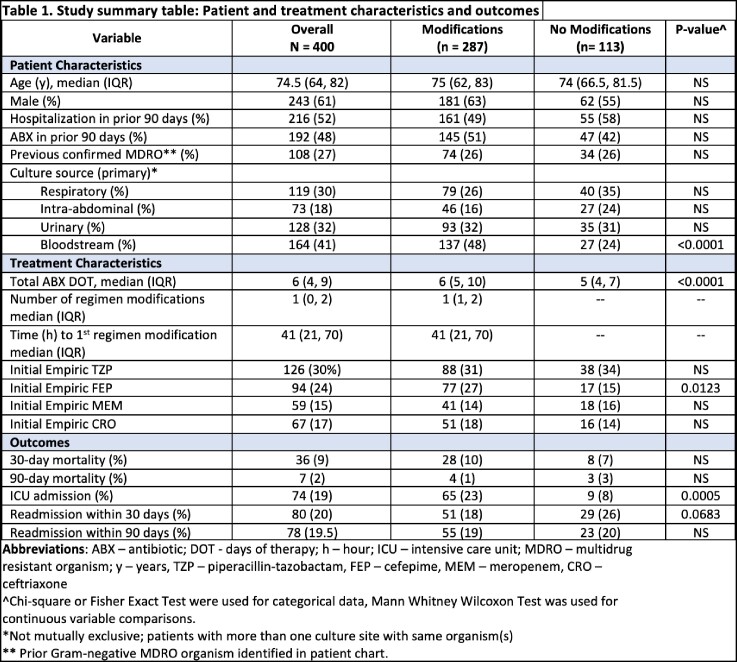

**Methods:**

This retrospective chart review included patients, admitted from 1/1/2019-12/31/2020 to Hoag Memorial Hospital, with a complicated GNBI due to an extended spectrum cephalosporin resistant pathogen. Patient charts were reviewed for demographics, antibiotic (ABX) modifications, rationale and timing of modifications, and patient outcomes. ABX modifications were defined as GNB ABX switched, added, or removed.

**Results:**

The study cohort included 400 patients (median age 74.5 years; 61% male). The predominant sources were lung (34%) and bloodstream (26%) for single cultures and bloodstream (33%) and urine (26%) for multiple cultures. The most common pathogens were ESBL organisms (36% overall with *E. coli* accounting for 81% of cases), and *Pseudomonas spp.* (40%). Inpatient initial empiric therapies included piperacillin-tazobactam (32%), cefepime (24%), meropenem (15%), and ceftriaxone (17%). ABX regimens were modified up to 6 times in 72% of patients. Median [IQR] time to first modification was 41 [21, 70] hours. For initial modifications, 70% were escalations, and most empiric modifications were based on preliminary culture results (56%) and lack of patient response or decompensation (25%). No difference in mortality and readmission was observed based on presence of ABX modifications.

**Conclusion:**

Many patients experienced modification to GNB empiric and targeted antibacterial regimens. In almost all cases, rationale for change was documented. No difference in patient outcomes was observed. Despite changes to ABX regimens, patient outcomes are not improved possibly due to shortcoming of existing antibiotic portfolio. Furthermore, the numerous changes in therapy observed may reflect the lack in identifying patients with resistant GNB organisms early in admission. This highlights the needs of more effective and novel antibiotics and importance of identifying patients at risk for resistant GNBI.

**Disclosures:**

**Jason Yamaki, PharmD, PhD**, Merck & Co., Inc.: Grant/Research Support **Emre Yucel, PhD**, MERCK: I am a full time Merck Employee and own stocks in the retirement plan provided by Merck.|MERCK: Stocks/Bonds **Alexandre H. Watanabe, PharmD**, Merck & Co., Inc.: Employee

